# A Risk Reduction Program for Osteoporosis Complications among Postmenopausal Women: A Quasi- Experimental Design

**DOI:** 10.1186/s12905-025-04013-6

**Published:** 2025-10-13

**Authors:** Fatin Abdel Raheem Mostafa Zewail, Faten Khayrat El-Guindi, Ferial Fouad Melika

**Affiliations:** 1 Staff nurse at an international educational hospital, Faculty of Nursing, Ain Shams University, Cairo, Egypt; 2 Professor of Family and Community Health Nursing, Faculty of Nursing, Ain Shams University, Cairo, Egypt

**Keywords:** Osteoporosis, Postmenopausal women, Risk factors

## Abstract

**Background:**

Osteoporosis is considered a common health problem in postmenopausal women. Therefore, reducing modifiable risk factors through dietary, lifestyle changes, and compliance of pharmacological therapy for those women at a significant risk of osteoporosis or fracture are essentials to the management of skeletal health of postmenopausal women.

**Purpose:**

The study aimed to evaluate the effect of risk reduction program of osteoporosis among post-menopausal women. The study hypothesizes that the risk reduction program will improve women’s knowledge and performance toward decrease complications of osteoporosis.

**Methods:**

A Quasi experimental research design was utilized to fulfill the aim of this study. The study was conducted at outpatient of osteoporosis clinic in Tanta university hospital, and Al-Minshawi general hospital. A purposive sample was used in this study of 126 women. The data gathered using three tools. Pretest done to assess postmenopausal women’s' knowledge, risk factor assessment tool was used to assess postmenopausal women’s practices, and medical history of postmenopausal women. The study was carried out over a seven-months period, commencing in early October 2023 and beginning of May 2024. The obtained findings were analyzed by appropriate statistical methods and tests of significance, data were presented in tables and charts using SPSS version 22 the statistical significance and associations were assessed using percentage values (%), mean value, and standard deviation (SD), and Chi square test (X^2^) and P- value, r test, and t-test.

**Results:**

According to this study there were 61.9% of postmenopausal women their age ranged between 55: ≥ 60 years with Mean ± SD = 54.80 ± 3.499, and there was improvement in total knowledge of postmenopausal knowledge as in preprogram was 13.20± 4.351 and improved to 25.85± 3.992 post program with a statistically significant difference (p<0.001), and there was improvement in total knowledge of postmenopausal knowledge as in preprogram was 92.9% had in adequate level of practice while after program became 74.6% had adequate level of practice regard prevention of complication of osteoporosis with a statistically significant difference (p<0.001).

**Conclusion:**

The risk reduction of osteoporosis program was effective and beneficial for improving postmenopausal women’s knowledge and practices regarding risk reduction of osteoporosis. Continuous of various educational programs for postmenopausal women should be implemented in the outpatient clinics in the hospitals through professional development programs.

## Background

Osteoporosis is a systemic skeletal disease characterized by low bone mass and microarchitecture-related bone tissue degeneration, which increases bone fragility and fracture susceptibility. According to the National Institute of Health (NIH), osteoporosis is a bone disease that results from a decrease in bone mass and bone mineral density (BMD). It is characterized by changes in the structure and strength of the bone and has a noticeable negative impact on the body, mind, society, and entire economy. The T score of a BMD scan can be used to diagnose osteoporosis [1]. Specifically, a family history of hip fractures is one of the risk factors for osteoporosis. In addition, age is a major risk factor for osteoporosis, as is female sex, because postmenopausal women are more likely to develop this condition. Reduced peak bone mass can be caused by inadequate calcium and vitamin D intake throughout growth. Women’s lifestyle choices, such as smoking, heavy alcohol use, and inactivity, can further increase the risk of osteoporosis [2]. The first stage of osteoporosis prevention should start in childhood, with an emphasis on a healthy diet and a variety of physical activities (PAs). Disease management and prevention strategies include leading a healthy lifestyle, receiving enough calcium and vitamin D, and controlling health conditions. Pharmaceutical treatments are another option for treating osteoporosis, and fall prevention strategies must be taken into account for women with osteoporosis who are at increased risk of falling. Exercise is essential for maintaining bone health and treating and preventing osteoporosis [3]. In women experiencing primary ovarian failure due to apoptosis or programmed cell death, menopause is a physiologically normal condition that occurs naturally. As people age, their ovaries typically operate less well. Follicle-stimulating hormone (FSH) levels increase, whereas estradiol synthesis decreases with the onset of menopause [4]. As ovarian function decreases with menopausal age, less estrogen is produced, and FSH levels rise in tandem. Osteoporosis in postmenopausal women begins with a period of rapid bone loss and considerable augmentation of bone resorption, which are caused by the combined effects of estrogen deficiency and increased FSH production. Postmenopausal osteoporosis may be crippling for people, but it is treatable in pharmaceutical and nonpharmacological ways [5]. Community health nurses play a vital role in encouraging women in the community to take proactive steps to prevent osteoporosis by holding osteoporosis screening camps; periodically assessing body math index (BMI), calcium, vitamin D, and bone mineral density levels; and educating women about lifestyle modifications such as regular exercise, sun exposure, maintaining a healthy weight, eating foods high in calcium, and taking calcium and vitamin D supplements. They should also assist women in making the right medication choices, such as calcium supplements combined with minerals, to prevent osteoporosis [6]. Attaining, regaining, and maintaining the general health of women and their families in the community through counseling is a key objective for community health nurses. When nurses are knowledgeable, well prepared, and educated, they are a significant important source of health information because their patients provide them with important information and present the public with primary and secondary prevention training regarding osteoporosis and its prevention [7]. Osteopenia affects 53.9% of postmenopausal Egyptian women, and osteoporosis affects 28.4%. Over 200 million individuals are estimated to be affected by osteoporosis globally; over 12 million Americans are affected, with postmenopausal women being more at risk, according to the International Osteoporosis Foundation. Aging women experience a complex interplay of factors that can contribute to abnormal osteoclast activation and increased bone resorption, such as decreased estrogen levels, a more sedentary lifestyle, chronic inflammation, and other related conditions [8]. In several studies aimed at preventing osteoporotic fractures, various educational intervention programs have been established to improve osteoporosis prevention and treatment through improved knowledge and practice, and these programs have been shown to be effective and improve womens’ knowledge and practices toward osteoporosis [9, 10] by addressing the risk factors for osteoporosis and have shown that unhealthy lifestyles are the main risk factors for decreasing bone mass in women with osteoporosis [11]. There are studies that discuss the benefits of preventive and remedial strategies for osteoporosis, including being physically active, providing adequate nutrition (distinctly calcium and vitamin D), and preventing unhealthy habits that have negative repercussions on bone health (such as tobacco use and alcohol intake), which are considered the most effective nonpharmaceutical strategies for preventing osteoporotic fracture especially in postmenopausal women [12]. To the best of our knowledge, there is limited research in Egypt focused on evaluating postmenopausal women’s knowledge and practices related to osteoporosis prevention. Therefore, the study hypothesized that a risk reduction program would improve postmenopausal women’s knowledge and practices toward decreasing complications of osteoporosis through modifying their life habits.

### Aim of the study

The present study aimed to evaluate the effect of a risk reduction program for osteoporosis among postmenopausal women. The study hypothesizes that a risk reduction program will improve postmenopausal women’s knowledge and practices toward decreasing complications of osteoporosis through modifying their life habits.

## Methods

### Study design

A quasi experimental research design was utilized to fulfill the study objectives (a pretest–posttest one group). The study was carried out over a seven-month period, launched in early October 2023 and beginning in May 2024.

### Study setting

The study was conducted with outpatients of osteoporosis clinics at Tanta University Hospital and Al-Minshawi General Hospital in the Tanta government according to the schedule plan of the Tanta government because these hospitals are considered the largest hospitals in the Tanta governorate and cover the health care services of all the residents in Tanta city and all the villages around it.

### Study participants

A purposive sample was used in this study with the following inclusion criteria: postmenopausal women aged 45–60 years, women diagnosed with osteoporosis, and women without any chronic diseases, such as diabetes mellitus or cardiac disease. The sample size was estimated according to the sample size equation to be 126 women to achieve a power of 95% and a level of significance of 5% (two sided), assuming an improvement of 30%.

Zα = standard normal deviation for α = 1.9600.

Zβ = standard normal deviation for β = 0.8416.

B = (Zα + Zβ)2 = 7.8489.

C = (E/SΔ)2 = 0.0625.

N = B/C = 125.5820.

**Fig. 1:**
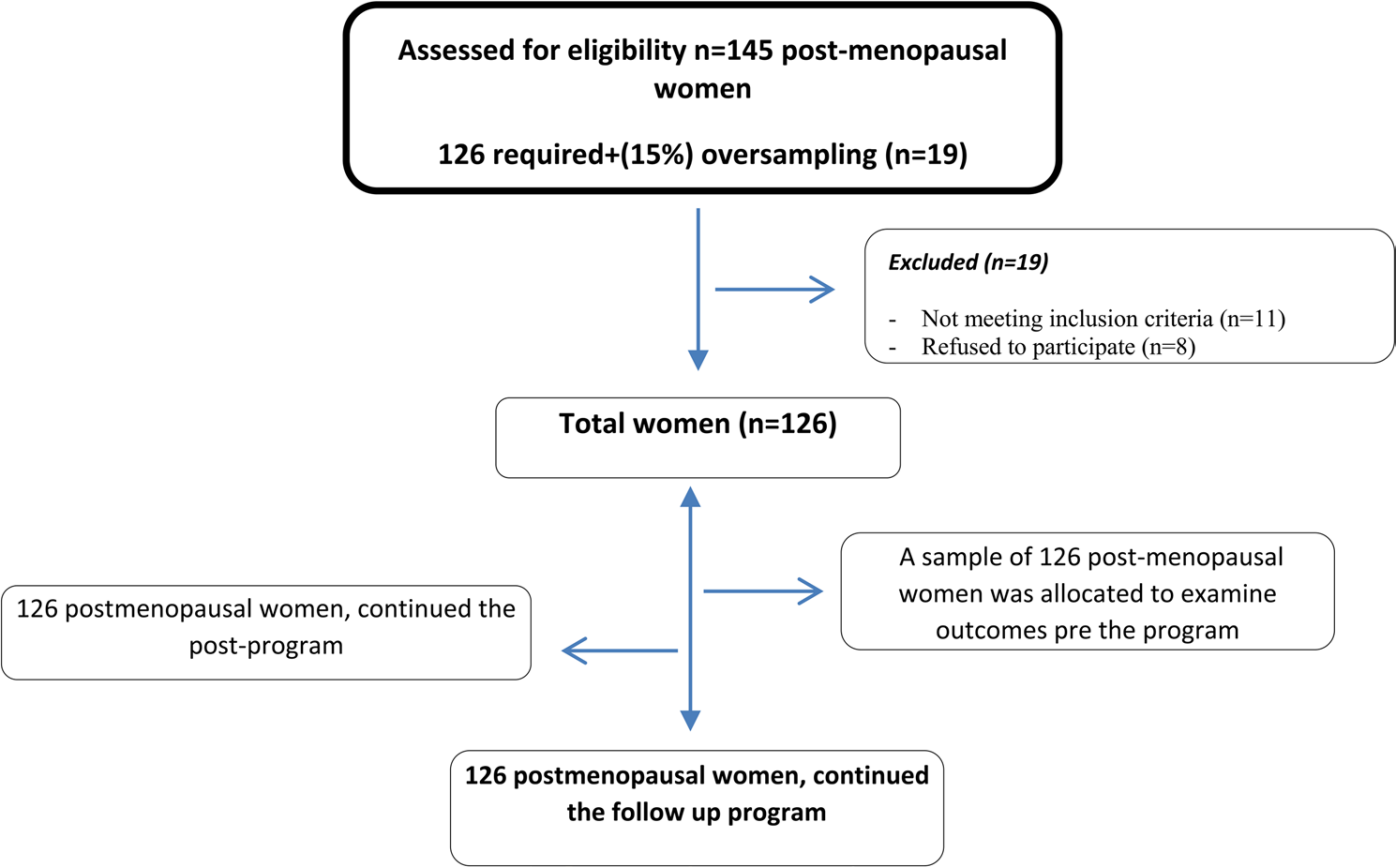
CONSORT flow diagram for postmenopausal women enrollment and follow-up

The N thus calculated is rounded up to the next highest integer to give the group size.

n= (1.96 + 0.84)/0.0625) ^2 = 125.5820 ≈ 126 women. We initially assessed 145 women (126+ (19)15% oversampling) to compensate for anticipated initial exclusions, 19 were excluded (11 didn’t meet inclusion criteria, 8 refused to participate), 126 women were allocated and all completed the study with no attrition as summarized in Fig. 1.

### Data collection instruments

Three tools were utilized in this study, namely, an interviewing assessment questionnaire, the medical records of postmenopausal women, and the physical data of postmenopausal women.

#### Tool (I)

The osteoporosis structured questionnaire was built by the authors and was guided by a review of relevant literature [13] to assess postmenopausal women’s knowledge and practices regarding osteoporosis throughout the program phases. It consists of four parts:

##### Part I

This part focused on collecting personal data of the study sample, such as age, education level, social status, residence, occupation, etc.

##### Part II

This part consists of closed-ended questions to assess postmenopausal women’s knowledge regarding osteoporosis, which are divided into seven main dimensions: (42) subitem questions; (2) questions related to the definition of osteoporosis as (osteoporosis is a thinning of the bones); (15) questions related to risk factors for osteoporosis, such as age, sex, race, the postmenopausal period, anddrinking soda; (6) questions related to signs and symptoms of osteoporosis, such as (back pain, stooped posture, and fracture can occur extremely easily); (3) questions related to complications of osteoporosis, such as (feeling of extreme pain, disability and inability to move…ect); (3) questions related to the diagnosis of osteoporosis, such as (DEXA scan, and laboratory investigations, such as Vit D, serum calcium levels….ect); (10) questions related to protective measures of osteoporosis, such as (exposure to sunlight, avoiding excessive caffeine and alcohol intake, maintaining regular exercise…ect); and (3) questions related to the treatment of osteoporosis, such as (hormone replacement therapy, bone-strengthening medications…ect). The postmenopausal women’s responses were examined via a model key answer set by the authors. For each question, a score of “one” was given for the right response; in addition, “zero” was given for the false response. The total level of knowledge was categorized as a satisfactory score of ≥ 50 or an unsatisfactory score of >50.

##### Part III

Risk factor assessment tool; the instrument was developed by a review of the relevant literature [13] and modified by the authors to assess risk factors related to osteoporosis. It was divided into five dimensions: (21) items; (7) questions related to nutritional patterns (eating food rich in calcium, drinking milk at least two cups daily); (3) questions related to follow-up (taking calcium supplements); (2) questions related to smoking habits (avoiding smoking); (5) questions related to physical exercise (practicing exercise at least 3 days per week and exercising at least 30 minutes each time); and (4) questions related to sun exposure (exposure to sunlight regularly, exposure for at least 15 minutes). The subjects’ responses were measured on a three-point Likert scale ranging from “never” with a score of“one” to “always” with a score of “three.” The scores of the items were summed, and the resulting score was divided by the number of items, giving a mean score for each dimension.

##### Part IV: Medical history of postmenopausal women

The instrument was developed by [19] and modified by the authors; it consists of two parts: postmenopausal women’s obstetric history, such as age at first menstruation, age at menopause, age at first pregnancy, number of years of menopause, number of pregnancies, and other information about postmenopausal women’s current complaints, such as feelings of pain.

#### Tool (II)

The physical sheet consisted of three questions to assess the woman’s weight, height, and BMI, with a BMI scoring system according to the World Health Organization (WHO) [14], and it was classified as normal weight (18.5–25), overweight (25–30), or obese (30 or above).

#### Tool (III)

**Fig. 2:**
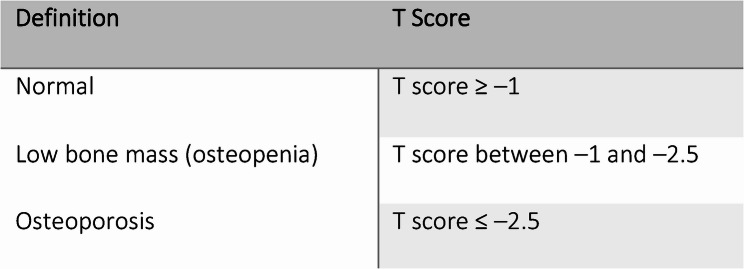
Classification of the degree of osteoporosis

In the medical records, the instrument was used to assess postmenopausal women’s laboratory investigations, radiological examinations (DEXA scan), and classification, as shown in Fig. (2).

### Instrument validity and reliability

First, the authors developed and modified the study instruments on the basis of a review of the literature, after which they were translated into Arabic. Later, a jury group evaluated the face and content validity of the instruments. This group included 3 expertise professors in community health nursing, Faculty of Nursing affiliated with Ain Shams University. The face and content validity sheet involved two parts: face validity included the opinions of the specialists about the general appearance, format, and understanding of the instruments and accuracy of language, whereas content validity covered the opinions of specialists about the importance, applicability, and relevance of the items. According to their opinions, the necessary modifications were made. Additionally, the instruments were examined for internal consistency (reliability) by employing Cronbach’s alpha coefficient. The osteoporosis knowledge questionnaire had good reliability (0.82), and the risk assessment tool had good reliability (0.86).

### Pilot study

It was conducted on postmenopausal women to evaluate the feasibility, applicability of tools, content, clarity of included questions, and simplicity, and then the necessary modifications were performed according to the results of the pilot study. This study was conducted on twelve postmenopausal women, representing 10% of the total sample (126 women). It took approximately one month from the beginning of September 2023, the time needed to fill out the tool was 30 min, and the appropriate modification was performed so that the subjects included in the pilot study were excluded from the actual study sample.

### Ethical consideration

The Research Ethics Committee (REC) of the Faculty of Nursing/Ain Shams University in Cairo city, Egypt, approved this study (code number 24.03.244) on the basis of the standards of the committee. The Dean of the Faculty of Nursing/Ain Shams University sent official letters to Tanta University Hospital and Al-Minshawi General Hospital in Tanta government city/Egypt, requesting their approval and collaboration for executing the study and gathering the data. These letters clarify the study’s purpose and procedures. Formal consent was received from each woman after explaining all the study phases and being instructed about her right to leave the study without giving rationales. The anonymity and confidentiality of the data gathered from the postmenopausal women were guaranteed.

### Fieldwork

The actual fieldwork for this study continued from the beginning of early October 2023 until the beginning of May 2024; it took seven months. The following five phases were used to implement the program:

#### First phase (Assessment phase)

This phase lasted one month from the beginning of October until the beginning of November 2023. After receiving ethical approval, the study was executed. The researcher then met with all the postmenopausal women to clarify the aim of the study and obtain consent from them for their participation in the study. The researcher then distributed the instruments to all the postmenopausal women to assess their knowledge and performance of osteoporosis complication prevention before program implementation. Later, the study instruments were personally collected and coded by the researcher. The collected data were considered pretest data, so they were analyzed by the researcher to assess women’s needs related to osteoporosis. Each woman took 30–40 min to complete the instrument.

#### Phase II (Planning phase)

This phase lasted one month, from the beginning of November 2023 until its end. On the basis of the results obtained from the data analysis in the assessment phase and a review of related literature, the researcher designed an osteoporosis program for postmenopausal women. Through the planning phase, the general aim of the program was set. The program aimed to improve women’s knowledge and performance toward decreasing the incidence of complications associated with osteoporosis. Additionally, the program plan was set, and methods of teaching and media were determined. In addition, the settings in which the program sessions will be were well equipped. Additionally, the program content, which included the theoretical and practical aspects of osteoporosis, was developed.

#### Phase III (Implementation phase)

This phase lasted two months, from December until the beginning of February 2024. During the implementation phase, the researcher divided the postmenopausal women into six groups, each consisting of twenty-one women. Every two groups attended one session per day, so the six groups attended through 2–3 days per week for a total of 8 weeks. Each session lasted for two hours, from 10 a.m. to 12 p.m. Additionally, the content of the program was presented through 5 sessions. Thus, the time allowed for the program was 5 h (4 theoretical hours and 1 practical hour). At the beginning of the 1 st session of the program, an orientation to the osteoporosis program, the aims of the program, the importance of the program, and the content of the program were clarified by the researcher. An introduction to the previous session was given before starting the next session, and its objectives were clarified as showed at Table 1. The program was implemented via various learning strategies, such as group discussions, lectures, and brainstorming. Additionally, educational media, such as data shows and videos, were used. Through the implementation of the program, different evaluation methods, such as a pretest and posttest, were used to assess the effect of the session. The researcher distributed the handout of the program to be used as a memorial reference for postmenopausal women.

**Table 1:** Osteoporosis program content

No	Sessions	Content
1	Introduction to osteoporosis.	Opening the program, importance of the program, prevalence of osteoporotic women, explain bone structure, and concept of osteoporosis.
2	Causes and risk factors for osteoporosis.	Recall information in the previous session through group discussion, identify risk factors for osteoporosis, and classified into modifiable and non-modifiable.
3	Diagnosis and screening of osteoporosis.	Recall previous information through questions and answers, explaining importance of diagnosis, the different methods for diagnosis as DXA, and serum calcium level, vitamin D level.
4	Preventive strategies.	Recall information through group discussion, demonstrating, and stressing on preventive behavior regard osteoporosis prevention through healthy lifestyle as nutritional habits and highlight the importance of food containing calcium and vit D, exposing to sunlight, finally explain suitable environment for fall prevention, and reducing hazards related to fall therefore fracture prevention.
5	Preventive strategies through practical measures.	Group discussion about the program, teach women types of exercise and how to apply, duration of exercise, apply how to expose their body to sunlight.

#### Phase IV (Post program evaluation phase)

This phase lasted one month through February 2024. The effects of the program on postmenopausal women’s knowledge of osteoporosis and performance of osteoporosis prevention were immediately evaluated after the end of the program through the posttest. The posttest was implemented by using the same instruments used in the pretest.

#### Phase V (Follow-up phase)

This phase lasted one month after 3 months of posttest to examine the program’s impact again through May 2024. The follow-up was implemented by using the same instruments used in the pretest. The data collection procedure in this phase was applied in the same manner as in the assessment phase and postprogram evaluation phase.

### Statistical analysis

The obtained findings were analyzed via appropriate statistical methods and tests of significance and then presented in tables. The collected data were organized, categorized, tabulated, and analyzed. The data are presented in tables and charts via SPSS (the Statistical Package for Social Science for Windows) version 22. Statistical significance and associations were assessed via percentage values (%), mean values, standard deviations (SDs), chi-square tests (χ^2^), P values, r tests, and t-test. Finally, confirmatory factor analysis was used for the adapted instruments to assess the relationships between the items and their dimensions, and a paired sample t-test of significance was used when comparing related samples. Differences were considered statistically significant for all the statistical tests if the p value was ≤ 0.05 and highly significant if the p value was *P* < 0.01.

## Results

### Distribution of the studied sample of postmenopausal women according to their demographic data.

As shown in Table 2, 61.9% of the postmenopausal women were between 55 and ≥ 60 years of age, with a mean ± SD = 54.80 ± 3.499. Additionally, 46.0% of women were uneducated, followed by 60.3% who were married and 57.9% who were not working with a monthly income of 500–2000 L.E.

**Table 2:** Distribution of the studied sample of postmenopausal women according to their demographic data (*n* = 126)

Demographic characteristics		No	%
Age	45: ˂ 50	10	7.9
	50: ˂55	38	30.2
	55: ≤ 60	78	61.9
	**Mean ± SD**	**54.80 ± 3.499**	
Level of Education	Uneducated	58	46.0
	Reads and writes	15	11.9
	Intermediate education	23	18.3
	Higher education	30	23.8
Social status	Single	5	4.0
	Married	76	60.3
	Divorced	5	4.0
	Widow	40	31.7
Residence	Urban	76	60.3
	Rural	50	39.7
Occupation	Working	53	42.1
	Not Working	73	57.9
Family monthly income	500–2000	53	42.1
	2500–3500	73	57.9
Home crowding Index	Uncrowded (> 1)	15	11.9
	Crowded (1–2)	42	33.3
	Overcrowded (< 2)	69	54.8
Any family member suffered from osteoporosis	Yes	68	54.0
	No	58	46.0
Degree of kinship (*n* = 68)	First	59	86.8
	Second	9	13.2

### Effect of the risk reduction program on postmenopausal women

Through program implementation, Table 3 shows that the knowledge of postmenopausal women was significantly different from total knowledge about risk reduction for osteoporosis, in which 14.3% of women had satisfactory knowledge before the program but improved to 79.4% and 65.9% at the post program and the follow-up phases, respectively, with p values > 0.001.

**Table 3:** Comparison of postmenopausal women’s knowledge regarding osteoporosis across phases of the program (*n* = 126)

Total Knowledge	Inadequate		Adequate		T- test		T- test	
					Post VS Pre		Follow-up VS Pre	
	No	%	No	%	Test	*P* Value	Test	*P* Value
Pre	108	85.7	18	14.3				
Post	26	20.6	100	79.4				
Follow-up	43	34.1	83	65.9				
Total mean knowledge score					t = 41.743	0.000**	t = 36.806	0.001**
Pre	13.20 ± 4.351							
Post	25.85 ± 3.992							
Follow-up	23.25 ± 4.278							

**P= (highly significant)

Table 4 shows that there was a highly statistically significant difference between total practices related to risk reduction programs for osteoporosis through program implementation phases; 7.1% of women had adequate levels of practice before the program but improved to 74.6% and 47.6% at the post program and follow-up phases, respectively with p values > 0.001.

**Table 4:** Comparison of postmenopausal women’s practices regarding osteoporosis across phases of the program (*n* = 126)

Total practices	Inadequate		Adequate		T- test		T- test	
					Post VS Pre		Follow-up VS Pre	
	No	%	No	%	Test	*P* Value	Test	*P* Value
Pre	117	92.9	9	7.1				
Post	32	25.4	94	74.6				
Follow up	66	52.4	60	47.6				
Total mean practice score					t = 21.941	0.000**	t = 17.189	0.000**
Pre	11.47 ± 4.174							
Post	25.83 ± 5.855							
Follow-up	22.40 ± 5.576							

**P= (highly significant)

Table 5 reveals that there was a statistically significant improvement in the postmenopausal women’s mean score regarding risk factors of osteoporosis throughout both the posttest phase and the follow up phase in comparison with pretest phase, with p values > 0.000.

**Table 5:** Comparison of the risk factors for osteoporosis throughout the phases of the program (*n* = 126)

Risk factors	Pre	Post	Follow up	Post VS Pre	Follow up VS Pre
	Mean ± SD	Mean ± SD	Mean ± SD	T test *P* value	T test *P* value
Body Mass Index.	32.91 ± 6.98	32.91 ± 6.98	30.75 ± 5.60	0.000 1.000	4.281 0.000**
Vitamin D level.	16.38 ± 4.19	18.65 ± 5.27	26.27 ± 4.56	3.880 0.000**	4.927 0.000**
Serum calcium level.	7.04 ± 1.45	8.41 ± 2.05	8.94 ± 1.43	4.074 0.000**	5.173 0.000**
Eating foods containing calcium.	1.59 ± 0.71	1.90 ± 0.55	2.75 ± 0.12	3.569 0.000**	5.196 0.000**
Eating foods containing vitamin D.	1.50 ± 0.75	2.16 ± 0.42	2.10 ± 0.45	4.785 0.000**	3.662 0.000**
Avoiding Soda/caffeine drinks.	1.88 ± 0.56	2.50 ± 0.25	2.25 ± 0.38	2.590 0.012*	2.187 0.026*
Taking calcium supplements.	1.91 ± 0.54	2.37 ± 0.32	2.64 ± 0.18	2.352 0.024*	2.645 0.019*
Taking vitamin D supplements.	1.33 ± 0.83	2.33 ± 0.34	2.56 ± 0.22	7.447 0.000**	9.048 0.000**
Passive smoking avoidance.	1.56 ± 0.72	2.20 ± 0.40	2.58 ± 0.21	6.354 0.000**	8.409 0.000**
Practicing Exercise.	4.79 ± 1.98	9.82 ± 2.53	8.14 ± 2.19	38.158 0.000**	27.372 0.000**
Exposure to Sunlight.	5.78 ± 2.7	4.32 ± 1.51	3.52 ± 1.82	5.023 0.000**	7.438 0.000**
**Total**	6.97 ± 1.95	7.96 ± 1.87	8.41 ± 1.56	4.337 0.000**	5.197 0.000**

## Discussion

The investigation of outcomes related to postmenopausal women’s knowledge of osteoporosis revealed that there was a statistically significance improvement in all dimensions of knowledge throughout the posttest phase and the follow up phase that compared with the pretest phase were had unsatisfactory level of knowledge. In Spain, these findings aligned with those of Senthilraja et al., [15], who reported that the studied women had a poor level of knowledge with respect to osteoporosis awareness. In Jordan, these findings are also supported by Abu Khurmah et al., [16], who reported that the studied women had low levels of knowledge and awareness of osteoporosis. Our findings, which are supported by Chithra, 2023 regarding the knowledge parameter levels of postmenopausal women regarding osteoporosis after program implementation, proved that there was improvement in women’s knowledge of osteoporosis after the application of the program compared with the pretest level of knowledge, with a p value >0.001. In the Bangalore Chithra study, there was improvement in women’s knowledge of osteoporosis after the application of the program compared with the pretest through the overall knowledge score in the pretest and in the posttest in the follow up, which was significant at the 5% level [17]. Poor knowledge of osteoporosis among postmenopausal women may be related to poor access to educational programs that specifically address osteoporosis and its risk factors. Additionally, most women may not have the ability to understand health information related to their educational condition, which makes it difficult for them to recognize the importance of the prevention of osteoporosis.

Additionally, the study by Elsyad& Ezzat, 2022, also supported our findings and revealed improvements in women’s knowledge of osteoporosis after program implementation compared with the pretest level of knowledge, with a p value of >0. 001 [18].

According to postmenopausal women’s practice toward osteoporosis complication prevention, it was clear from these findings that there was a statistically significance improvement in postmenopausal women’s practices throughout the posttest phase and the follow up phase in comparison with the pretest phase that the women had un satisfactory level of practice with a p value < 0.001. In Ankara, our findings were in accordance with those of Saltık et al., [19], who reported that half of women had a low level of prevention of osteoporosis complications. In Nepal, Khanal et al., [20] contrasted with our study findings and reported that half of women had good practice regarding prevention of osteoporosis complications. The inadequate practice of osteoporosis prevention among postmenopausal women in pretests may be related to a lack of coordination among healthcare providers, which can result in missed opportunities for education, as the importance of physical activity for bone health can lead to a sedentary lifestyle in which the risk of osteoporosis and its complications are increased. In Iran, research by Shams et al., [21] and Salimi et al., [22] supported our findings and revealed improvements in women’s practices with respect to preventing osteoporosis complications posttest with a p value < 0.001. Additionally, in Egypt, our study findings concurred with those of Ibrahim et al., [23], who reported that there was a statistically significant relationship between women on the posttest after program implementation, with a p value < 0.001. The improvement in women’s knowledge and practices in our study may be related to the wide variety of educational methods used, such as audiovisual materials, videos, lectures, and discussions, as well as the Arabic booklet distributed at the end of the sessions to be available to them everywhere and every time.

Regarding comparison of the risk factors related to osteoporosis throughout the phases of the program, in this study, there was a statistically significant improvement in all items related to osteoporosis risk factors as perceived by postmenopausal women throughout the posttest phase and follow up phase in comparison with the pretest phase. These statistically significant improvements were attributed to the positive effect of the risk reduction program.

In Egypt, Hassanine et al., [24] largely supported these findings, and Jo et al., [25] who reported a statistically significant improvement in risk factors regard osteoporosis with p value > 0.001. In Turkey, the findings agreed with those of Kalkım& Dağhan, [26], who discussed osteoporosis prevention education and counseling program for women and reported that there was a significant improvement in women practice related to daily exercise and calcium intake in relation to program implementation, with a p value >0.05. Additionally, in Iran these findings are accepted by Najafi et al., [27], and Pakyar et al., [28] who were interested in the effect of educational intervention on preventing osteoporosis in postmenopausal women and reported improvements in postmenopausal women’s healthy habits, such as engaging in physical activity, drinking tea or coffee, avoiding active or passive smoking, and exposure to sunlight, with p values < 0.001. Finally, the educational program was beneficial for postmenopausal women and improved their knowledge and practice after the pretest, but there was also a decrease in the follow-up score related to the long-term period for reassessing their knowledge and practice, which clarifies the need for continuous education for women.

### Limitations of the study

Despite the positive results of the study, there are limitations to consider. There is a limitation of previous studies related to postmenopausal osteoporosis. Thus, there is a strong need for more studies on this topic to foster and generalize the current findings. Additionally, the data were self-reported, which may limit the generalizability of the study results because of the risk of bias, and not all health care providers in outpatient clinics have enough knowledge to provide osteoporotic women with enough information with respect to osteoporosis complication prevention. In addition, the long-term effect of the educational program is not very beneficial for women because of the lack of continuous follow-up for women; thus, continuing education and follow-up is needed to improve the knowledge and practices of postmenopausal women with respect to osteoporosis and educating health care providers to provide osteoporotic women with enough information for improving their lifestyle and continuous screening to control complications. Finally lack of control group may limit the results of the study, so it recommended for effective evaluation of the program intervention.

## Conclusion

In conclusion, there was a highly statistically significant improvement in postmenopausal women’s knowledge and practices regarding risk reduction for osteoporosis complications through both the posttest phase and the follow-up phase in comparison with the pretest phase. Additionally, it was a highly statistically significant improvement in healthy lifestyle habits among women resulting in a decrease in osteoporosis risk factors that prevent its complication. In light of the findings of this study, it was concluded that the risk reduction of osteoporosis program was effective and beneficial in improving postmenopausal women’s knowledge and practices regarding the risk reduction of osteoporosis complications.

### Implications for practice

This research provides valuable recommendations that can impact effective strategies for good bone health in women. Conclusively, these findings can lead to improvements in postmenopausal women’s awareness and practices with respect to osteoporosis complication prevention through a multifaceted approach that includes diet, exercise, and lifestyle changes and encourages women to embrace the importance of osteoporosis management. In addition, the results will lead to the development of best practice guidelines for healthcare providers, offering evidence-based recommendations for osteoporosis complication prevention, specifically for postmenopausal women. 

## Data Availability

The datasets used and analyzed during the current study are available from the corresponding author on reasonable request.

## Abbreviations

NIH: National institute of health
REC: Research ethics committee
BMD: Bone mineral density
BMI: Body mass index
PA: Physical Activities
FSH: Follicle-stimulating hormone
